# Supplementary material for: Self-assembly of the smallest and tightest molecular trefoil knot
Source: Nat Commun. 2024 Jan 2;15:154. doi: 10.1038/s41467-023-44302-y (PMC10762025; doi:10.1038/s41467-023-44302-y)
Supplement: Supplementary file 1 — Supplementary Information [file 41467_2023_44302_MOESM1_ESM.pdf]

Supplementary Information

Self-assembly of the smallest and tightest molecular trefoil knot

Zhiwen Li,[1,2] Jingjing Zhang,[1,2] Gao Li,[1,2,*] and Richard J. Puddephatt,[3,*]

[1] State Key Laboratory of Catalysis, Dalian Institute of Chemical Physics, Chinese Academy of Sciences, Dalian 116023, China.

[2]University of Chinese Academy of Sciences, Beijing 100049, China

[3]Department of Chemistry, University of Western Ontario, London N6A 5B7, Canada

*Email: gaoli@dicp.ac.cn (G.Li); pudd@uwo.ca (R. J. Puddephatt)

I. Materials.

All chemicals, including solvents, were commercially available as reagent grade and used as received without further purification. $HAuCl_4 \cdot 3H_2O$ (98%), K_2CO_3 (95%), 3-bromoprop-1-yne (99%), pyrocatechol (98%), 1,4-bis(diphenylphosphino)butane, and Et_3N (99%) were purchased from Adamas-beta®. Ultrapure water was purified via a Barnstead Nanopure Di-water TM system. All glassware was thoroughly cleaned with aqua regia (37 wt% HCl: HNO_3 = 3:1 by volume), rinsed with copious ultrapure water, and then dried in an oven prior to use. 1,2-bis(prop-2-yn-1-yloxy)benzene **(**H_2L) ligand and L-Au_2 were prepared according to the reported method.[1]

II. Methods.

UV-visible spectra were measured using a Shimadzu UV-1800 spectrophotometer in CH_2Cl_2 solution. MALDI-TOF-MS was performed using an ABI MALDI TOF/TOF 5800 in a positive ion mode using trans-2-[3-(4-tert-butylphenyl)-2-methyl-2-propenyldidene]malononitrile as matrix material. ESI-MS was performed by using an electrospray PE-Sciex mass spectrometer as a solution in acetonitrile with NaOTf to aid ionization. IR spectra were recorded using a Bruker vertex 70 infra-red spectrometer (resolution 5 cm^{-1}, scan: 32, scale: 600-4000 cm^{-1}) as KBr disc. NMR spectra were recorded using an AVANCE III 400 MHz (Bruker) spectrometer. The DFT calculations were carried out using the BLYP functional, with double-zeta basis set and first-order scalar relativistic corrections. In each case several potential conformers were tested first by molecular mechanics minimization followed by DFT, with the lowest energy reported. The solvent effect of dichloromethane was modeled by using COSMO, all as implemented in ADF-2020.[2] Relative Gibbs free energies are given in Figure S9.

III. X-ray crystallography.

A suitable crystal was mounted on a XtaLAB AFC11 (RCD3) diffractometer (thf solvate) or Bruker APEX-II CCD diffractometer (CH_2Cl_2 solvate). Data reduction, cell refinement and experimental absorption correction were performed with the software package of CrysAlisPro. The structures were solved by intrinsic phasing methods by SHELXT 2018 and refined against F^2 by full-matrix least-squares by SHELXL 2018[3,4] All non-hydrogen atoms were refined anisotropically. Hydrogen atoms were generated geometrically. All calculations were carried out by the program package of Olex2 (ver 1.2.10.32).[5] Electron density due to disordered solvent molecules in the thf solvate was treated by using SQUEEZE.[6] Crystallographic data are given in Tables S1 and S2 and in the CIF file, which has been deposited at the Cambridge Crystallographic Data Centre, under deposition number CCDC 2278951. Copies of the data can be obtained free of charge via https://www.ccdc.cam.ac.uk/structures/.

IV. Synthesis of Au_6 knot

(a) Solvate 2**Au_6**.$2H_2O$.3thf. The L-Au_2 (10 mg, 0.017 mmol) was dispersed in thf (2 mL), followed by the addition of dppb (dppb = 1,4-bis(diphenylphosphino)butane) (7 mg) under rapid stirring. After 12 h., residual insoluble solid was removed by filtration, followed by vapour diffusion of diethyl ether into the filtrate to give colorless crystals of 2**Au_6**.$2H_2O$.3thf. Yield: 7.2 mg, 42%. MALDI-TOF-MS: m/z = 623.8 $[Au(dppb)]^+$, 1005.0 $[Au_2L(dppb)]H^+$, 1201.0 $[Au_3L(dppb)]^+$. IR: ν(C≡C) = 2142 cm^{-1}. NMR in CD_2Cl_2 (**Au_2**): δ(^1H) = 2.10 (m, 4H, CH_2C), 2.23 (m, 4H, CH_2P), 4.78 (s, 4H, CH_2O), 6.75 (m, 2H, C3,C6, catechol), 7.05 (m, 2H, C4,C5, catechol), 7.3 (m, 12H, H*o*,H*p*, PhP), 7.6 (m, 8H, H*m*, PhP); δ(^{31}P) = 39.2 (s).

(b) Solvate **Au_6**.$3CH_2Cl_2$. The L-Au_2 (10 mg, 0.017 mmol) was dispersed in CH_2Cl_2 (2 mL) followed by the addition of dppb (dppb = 1,4-bis(diphenylphosphino)butane (7 mg) dissolved in CH_2Cl_2 (2 mL) dropwise under rapid stirring. After 12 h at room temperature, the residual solid was removed by filtration. Colorless crystals of **Au_6**.$3CH_2Cl_2$ were obtained by the diffusion of diethyl ether into the filtrate for two weeks at 4°C in a refrigerator. Yield: 3.5 mg, 18%. ESI-MS (MeCN/NaOTf)): Calc. for $Au_2(L)(dppb)Na^+$, m/z = 1027.14; Found, m/z = 1027.20. Anal. Calc. for $C_{123}H_{114}Au_6Cl_6O_6P_6$: C 45.20, H 3.52. Found: C 45.31, H 3.80%.

V．Supporting figures

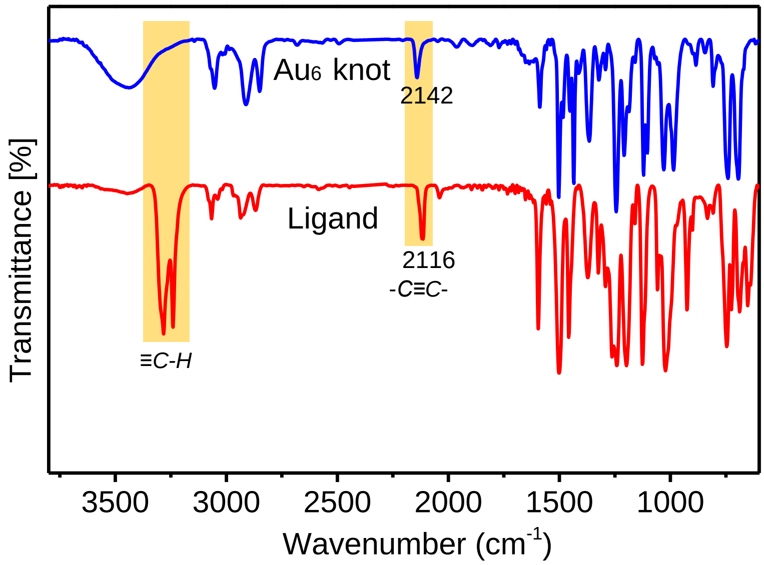

Supplementary Figure 1. IR spectra for H_2L and Au_6 knot (as thf solvate).

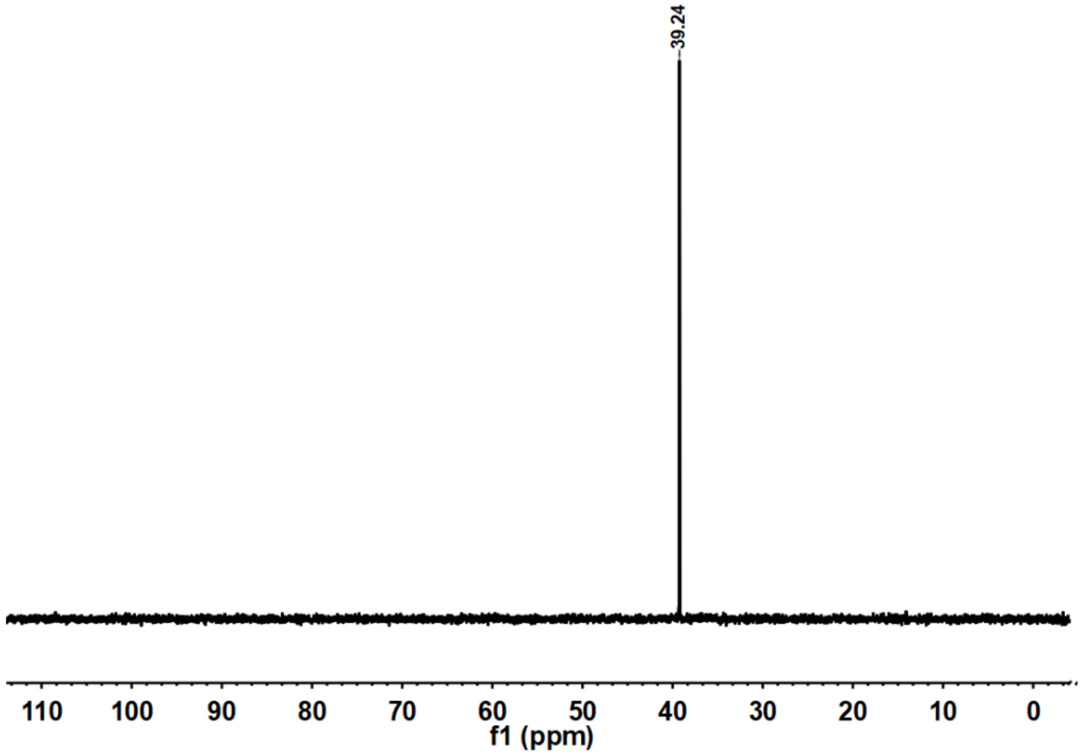

Supplementary Figure 2. ^{31}P NMR spectrum of Au_6 knot.

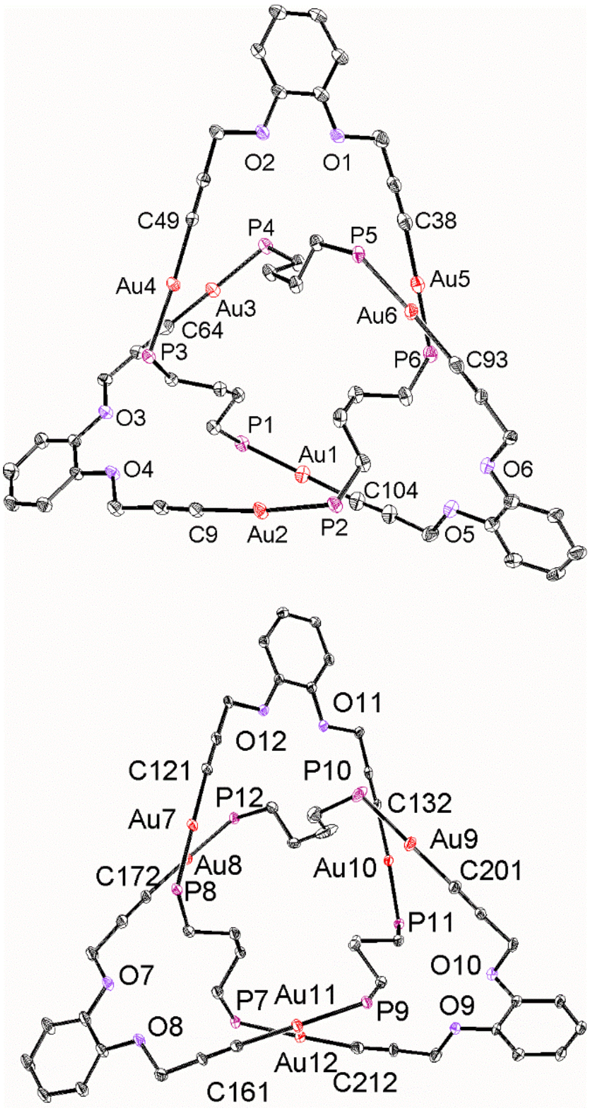

Supplementary Figure 3. The two non-equivalent molecules of **Au6**, in the thf solvate, showing the atom labels (phenyl groups and H-atoms are omitted, for clarity).

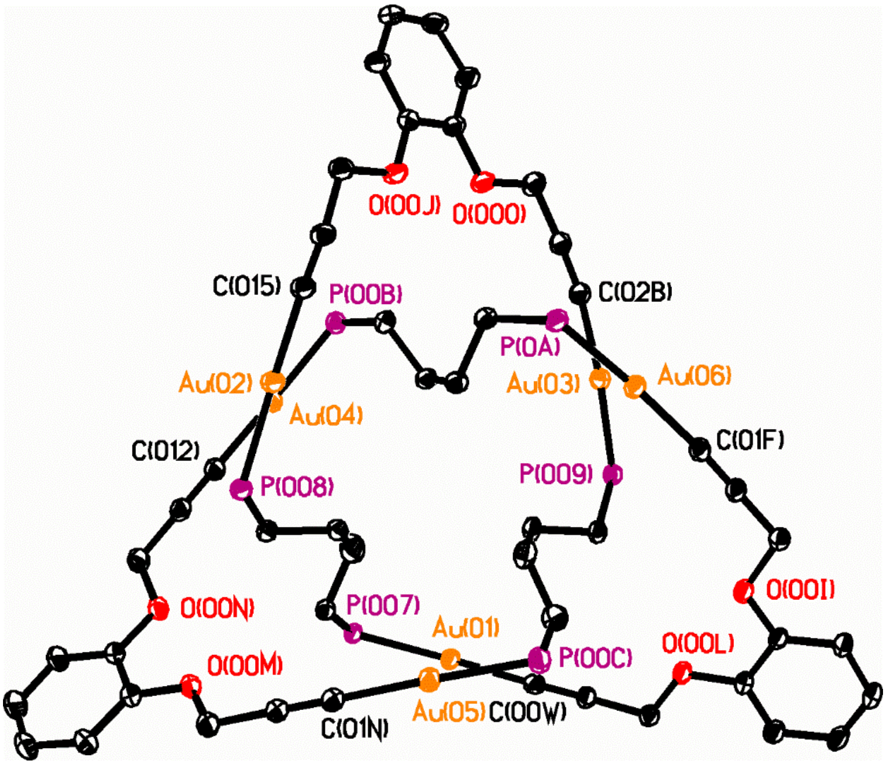

Supplementary Figure 4. The structure of **Au6** in the solvate **Au6**.$3CH_2Cl_2$, with the atom numbering scheme. Phenyl groups and hydrogen atoms are omitted, for clarity.

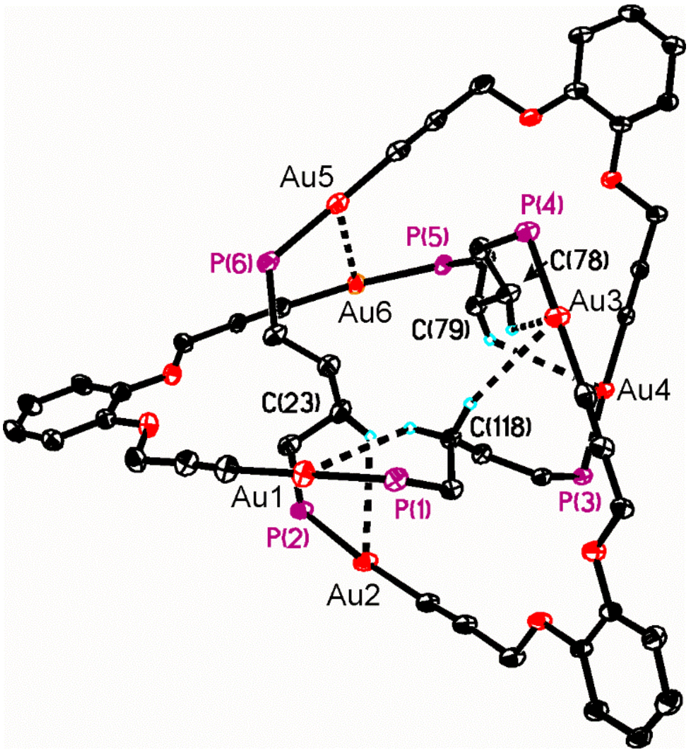

Supplementary Figure 5. Short contacts in the structure of molecule 1 of **Au6** as the thf solvate. Selected parameters: Au1..H118 3.07, Au2..H23 3.06, Au3..H118 2.96, Au3..H78 3.04, Au4..H79 3.07, Au5..Au6 3.68 Å. The van der Waals distances Au..Au and Au..H have been variously estimated in the ranges 3.32-4.28 and 2.75-3.34 Å respectively,[7,8] while aurophilic bond distances Au..Au and Au..H hydrogen bond distances are cited in the range 2.85-3.50 Å and approximately 2 Å, respectively.[9,10]

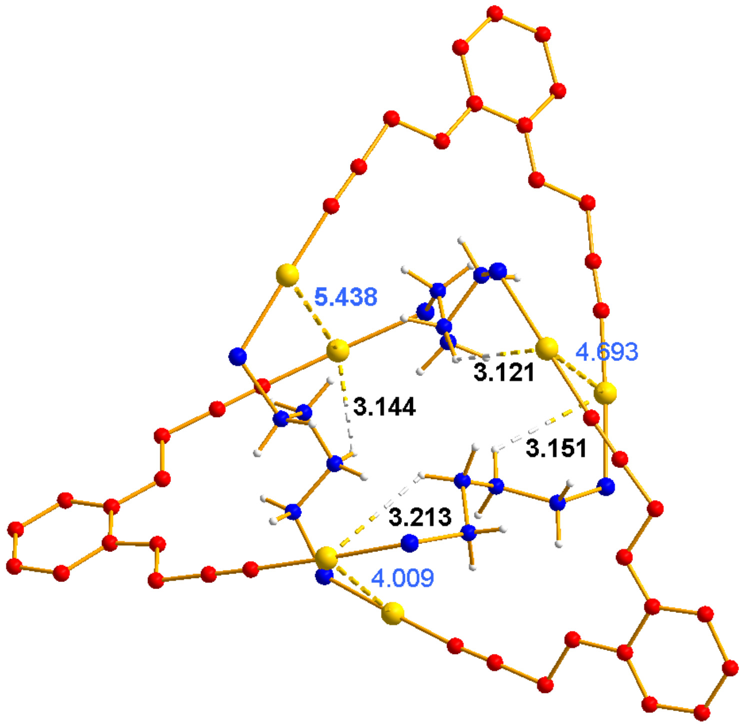

Supplementary Figure 6. The H---Au and Au---Au contacts in molecule 2 of thf solvate of **Au6**. The observed Au---H(CH_2) and Au---Au distances are in the range from 3.121 Å to 3.213 Å and 4.009 Å to 5.438 Å, respectively.

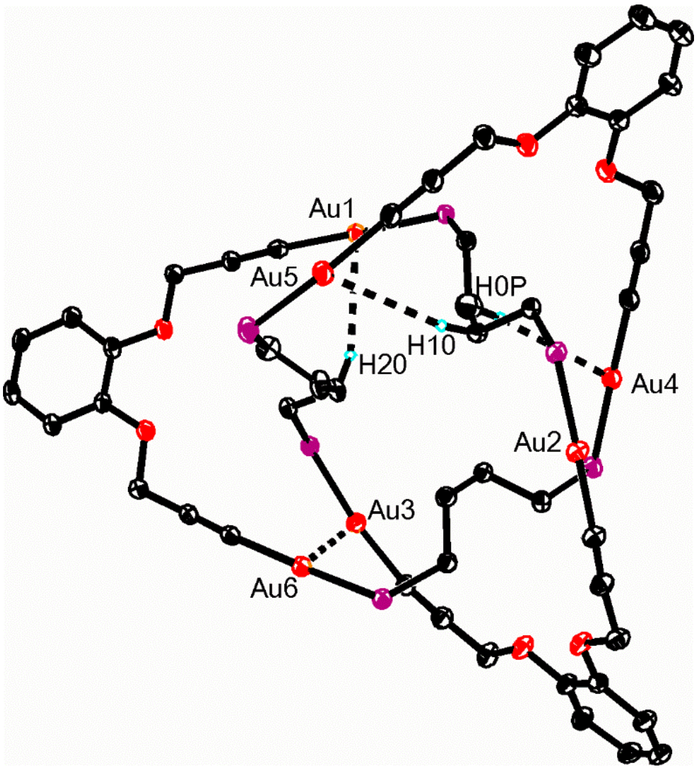

Supplementary Figure 7. Short contacts in the structure of **Au6** as the dichloromethane solvate. Selected parameters: Au1..H20 2.99, Au4..H0P 3.09, Au5..H10 3.03, Au3..Au6 3.44 Å; P-Au3-C 170.9, P-Au6-C 173.8.

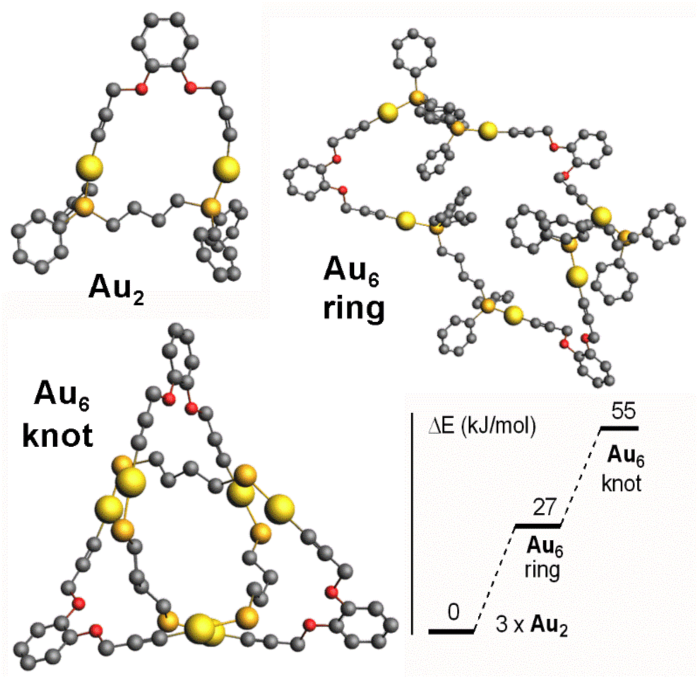

Supplementary Figure 8. Calculated structures of **Au$_2$**, **Au$_6$** (ring) and **Au$_6$** (knot) and relative Gibbs free energies. The phenyl groups are not shown for **Au$_6$** (knot), for clarity.

VI. Supplementary Tables

Supplementary Table 1

Crystallographic data for complex **Au_6**

formula_moiety	$2(C_{120}H_{108}Au_6O_6P_6).2H_2O.3(C_4H_8O)$	$C_{120}H_{108}Au_6O_6P_6$. $3CH_2Cl_2$
formula	$C_{252}H_{244}Au_{12}O_{17}P_{12}$	$C_{123}H_{114}Au_6Cl_6O_6P_6$
formula weight	6279.70	3268.45
crystal system	triclinic	monoclinic
space group	P-1	$P2_1/c$
cell dimens.		
a/ Å	17.74717(14)	28.2037(2)
b/ Å	27.1503(3)	22.8042(2)
c/ Å	28.8912(2)	18.3386(2)
$\alpha/^{o}$	114.7394(9)	90
$\beta/^{o}$	101.4684(7)	99.1100(10)
$\gamma/^{0}$	96.5471(7)	90
V/ $Å^3$	12082.8(2)	11645.93(19)
Z	2	4
T/K	100	293
R ($I>2\sigma I$)	0.0648	0.0386
wR2 (all data)	0.1744	0.0971

Supplementary Table 2. Selected bond parameters for **Au_6**.$2H_2O$.3thf

Molecule 1, Au1-Au6	Molecule 2, Au7-Au12
Au1 P1 2.274(3)	Au7 P8 2.274(2)
Au2 P2 2.273(3)	Au8 P12 2.284(2)
Au3 P4 2.271(3)	Au9 P10 2.271(3)
Au4 P3 2.277(2)	Au10 P11 2.280(3)
Au5 P6 2.285(4)	Au11 P9 2.280(2)
Au6 P5 2.284(3)	Au12 P7 2.283(3)
Au1 C104 2.034(15)	Au7 C121 2.023(10)
Au2 C9 2.016(12)	Au8 C172 1.996(10)
Au3 C64 1.974(14)	Au9 C201 1.979(11)
Au4 C49 1.994(10)	Au10 C132 1.987(11)
Au5 C38 2.013(16)	Au11 C161 2.006(10)
Au6 C93 2.027(14)	Au12 C212 1.998(13)
Au1 Au2 4.909	Au7 Au8 4.009
Au3 Au4 5.234	Au9 Au10 5.437
Au5 Au6 3.678	Au11 Au12 4.693
C104 Au1 P1 175.9(4)	C121 Au7 P8 177.7(3)
C9 Au2 P2 174.6(3)	C172 Au8 P12 176.9(4)
C64 Au3 P4 175.7(4)	C201 Au9 P10 174.3(4)
C49 Au4 P3 175.7(3)	C132 Au10 P11 174.5(3)
C38 Au5 P6 176.0(4)	C161 Au11 P9 176.0(3)
C93 Au6 P5 172.3(4)	C212 Au12 P7 176.9(4)

Supplementary Table 3.   Selected bond parameters for **Au₆**.3CH₂Cl₂

Au01 P007 2.2805(14)
Au01 C00W 1.993(7)
Au02 P008 2.2760(14)
Au02 C015 2.010(6)
Au03 P009 2.2763(15)
Au03 C02B 1.980(7)
Au04 P00B 2.2725(16)
Au04 C012 2.002(7)
Au05 P00C 2.2785(17)
Au05 C01N 2.011(7)
Au06 P0A 2.2775(16)
Au06 C01F 2.000(7)

C00W Au01 P007 176.05(19)
C015 Au02 P008 178.05(19)
C02B Au03 P009 170.9(2)
C012 Au04 P00B 177.1(2)
C01N Au05 P00C 172.0(2)
C01F Au06 P0A 173.8(2)

Au01 Au05   5.199
Au02 Au04   4.711
Au03 Au06   3.444

References

1. Shi, Q., Qin, Z., Ping, G., Liu, S., Xu, H. & Li, G. *RSC Adv.*, **10**, 21650–21655 (2020).
2. (a) Becke, A.D. *Phys. Rev. A* **38**, 3098-3100 (1988). (b) Andzelm, J., Kolmel, C., Klamt, A. *J. Chem. Phys.* 103, 9312-9320 (1995). (c) ADF 2020, SCM, Vrije Universiteit, Amsterdam, The Netherlands, http://www.scm.com. (d) Spek, A.L. *Acta Crysallogrt. C* . **71**, 9-18 (2015).
3. Sheldrick G.M. *Acta Crystallogr. A* **71**, 3 (2015).
4. Sheldrick, G.M. *Acta Crystallogr. C*. **71**, 3 (2015).
5. Dolomanov, O. V., Bourhis, L. J., Gildea, R. J., Howard, J. A. K. & Puschmann, H. *J. Appl. Crystallogr*. **42**, 339 (2009).
6. Spek, A.L. *Acta Crystallogr. C.* **71**, 9-18 (2015).
7. Bondi, A. *J. Phys. Chem.* **68**, 441-451 (1964)

8. Hu, S.-Z., Zhou, Z.-H., Robertson, B.E. *Z. Krist.* **224**, 375-383 (2009).

9. Schmidbaur, H., Schier, A. *Chem. Soc. Rev.* **41**, 370-412 (2012).

10. Schmidbaur, H. *Angew. Chem. Int. Ed.* **58**, 5806-5809 (2019).
